# An education-based intervention investigating the accuracy of community-based optometrists evaluating limbal anterior chamber depth

**DOI:** 10.1038/s41433-024-03492-5

**Published:** 2024-12-06

**Authors:** Anish Jindal, Tess Agnew, Dilani Siriwardena, Eleni Nikita, Winifred Nolan

**Affiliations:** 1 Moorfields Eye Hospital NHS Foundation Trust, London, UK; 2 Institute of Ophthalmology, University College London, London, UK; 3 NIHR Moorfields Biomedical Research Centre, London, UK

**Keywords:** Education, Health care

## Abstract

**Introduction:**

In the UK, referrals for suspected primary angle closure (PAC) by community-based optometrists (CBO) to the hospital eye service show moderate accuracy. This study aimed to assess the interobserver agreement of limbal anterior chamber depth (LACD) between CBOs and ophthalmologists and evaluate the impact of an education intervention.

**Methods:**

Using a 7-point LACD grading scale, consultant ophthalmologists graded an LACD image dataset developed from 100 patients attending Moorfields Eye Hospital. Two sets of 84 images were utilised for two LACD online assessments. CBO were recruited and undertook assessments before and after a PAC education package (EP) between August 2023 and January 2024.

**Results:**

Fifty-two optometrists completed the initial LACD assessment with a median of 19.0 (IQR 9.3–24.8) years post-registration experience. Using the 7-point LACD grading scale, mean weighted kappa (Kw) for pre-EP was moderate, 0.42 (95% CI, 0.36–0.48), increasing to 0.47 (0.42–0.53) post-EP. Conversion to the 4-point grading scale, Kw was substantial, pre-EP 0.61, (0.56–0.66) and post-EP Kw 0.64 (0.58–0.69). An LACD threshold (<25%) sensitivity and specificity pre-EP was 86.0% (79.9–91.3%) and 84.6 (82.3–86.9), respectively. Post-EP sensitivity decreased to 78.2% (74.1–85.1%) (*p* = 0.049) but specificity increased to 90.4 (88.1–92.8) (*p* < 0.001).

**Conclusion:**

The 4-point LACD grading scale would be more applicable for a case-finding setting. An accessible EP improved agreement and specificity using the joint Royal College of Ophthalmologists/College of Optometrists PAC referral threshold. This EP may reduce false positive PAC referrals and could be rapidly disseminated to CBO. Further research is needed to assess real-world PAC referrals after an education intervention.

## Introduction

Glaucoma is the leading cause of irreversible blindness worldwide. Globally primary open-angle glaucoma is more common than primary angle closure glaucoma (PACG) [1], but PACG is more likely to result in bilateral blindness and accounts for 50% of glaucoma blindness [2, 3]. Non‐contact tests have been developed to identify people at risk of angle closure, which are relatively quick to conduct and can be carried out by appropriately trained healthcare professionals in primary care, who can then refer onwards for specialist assessment [4].

In the UK, it has been found that the accuracy of referrals for suspected primary angle closure (PAC) by community-based optometrists (CBO) to the hospital eye service is low to moderate [5, 6]. Recent guidance on the management and referral of primary angle closure has been published by the Royal College of Ophthalmologists (RCO) and the College of Optometrists (CoO) [7, 8]. The guidelines recommend referring individuals with risk factors for developing PACG in their lifetime and a limbal anterior chamber depth (LACD) grading of less than 25%, to the hospital eye service. A meta-analysis of a LACD threshold of 25% or less yielded relatively high sensitivity and specificity [4], yet it has been found that both CBO interobserver agreement of LACD and application of RCO/CoO guidance is moderate [9, 10]. Furthermore, there is a risk that the inclusion of a LACD threshold in the guidelines could induce an increase in inappropriate referrals.

The primary objectives of this study were to determine the interobserver agreement of LACD between CBO and ophthalmologists using the traditional 4-point and 7-point grading scales [11, 12]. In addition, it was sought to evaluate the accuracy of CBO regarding LACD thresholds that are recommended by clinical management guidelines [7, 8, 13], as well as to determine whether an education intervention would affect agreement and measurements.

## Methods

A prospective study was conducted at Moorfields Eye Hospital (MEH) NHS Foundation Trust, City Road, United Kingdom between December 2022 and March 2024. The study was approved by the MEH Research Ethics Committee and complied with the tenets of the Declaration of Helsinki. IRAS number 315329. Ethics was approved on 11 August 2022, approval number 22/EE/0172. Clinical trials gov registration number NCT05543889.

### Case vignettes

An LACD image dataset was required to develop two online vignette assessments for CBO to undertake pre and post a primary angle closure disease education intervention. The LACD images were taken from patients who met the following inclusion criteria: they were 18 years or older and attended the new patient glaucoma clinic at MEH, City Road. Patients were excluded if they couldn’t be imaged using a table-mounted camera attached to a slit lamp due to physical limitations, if they had gross nystagmus or were unable to consent. Patients were screened for eligibility one month before their outpatient attendance and were then sent a study information leaflet. Consent was taken at their clinical appointment. Patient data was pseudonymised and their age and gender were recorded.

Each participant had their LACD captured using a slit lamp with an integrated photography unit (Model 900 BM, Haag-Streit), under constant dim room lighting conditions, by an experienced glaucoma optometrist. The illumination column of the slit lamp was offset at 60 degrees temporal to the microscope and a narrow-slit beam measuring 3 mm in height was positioned at the most peripheral point of the cornea, just inside the temporal limbus. Using ×16 magnification, an image was taken of each eye. All the images were reviewed independently for clarity and positioning and one image from each eye was then selected for grading by an independent expert panel.

### Expert panel

An expert panel of three consultant ophthalmologists sub-specialised in glaucoma graded each image. The width of the narrowest gap between the reflection of the beam on the iris and the corneal section was expressed as a percentage of the total thickness of the cornea. Each result was recorded using a seven-point percentage grading scale [12] (0%, 5%,15%, 25%, 40%, 75%, ≥100%) or as ungradable. All graders were masked to each other’s results. The grades were then collated independently by the principal investigator. The final grade was assigned for each image if either two or more grades were the same. If the grades differed across all 3 graders, a graticule was used to determine the percentage gap and its respective grade.

### Participating optometrists

CBO were recruited to undertake the assessments and education package. UK community-based optometrists were recruited through posters distributed and promoted electronically via Moorfields Eye Hospital, local optical committees and social media where an incentive of continued professional development (CPD) accreditation was offered for enrolment and completion of the study. Optometrists expressing an interest were asked to complete an eligibility questionnaire. To be included as participants they had to be registered in the UK and practising in the community for at least 2 days per week. Telephone consent was obtained from all participants and their information pseudonymised. They were then asked to complete an online questionnaire (Supplementary 1) through a secure link that was open to responses between July and September 2023 that asked both structured and open-text questions with reminders sent out monthly. The questionnaire was constructed using Research Electronic Data Capture [14, 15] (REDCap) and data was managed at Moorfields Eye Hospital and was reviewed by the public involvement lead at MEH.

### Assessment and education package

Development of the two online vignette assessments was constructed using Microsoft Forms. The case mix for the images used in both assessments was based on the average reported prevalence of narrow angles in a UK specialist setting [5, 6]. For each assessment, one image (eye) from each participant was utilised, none of the images from the first assessment were used in the second assessment, nor were any images repeated in either assessment.

**Fig. 1:**
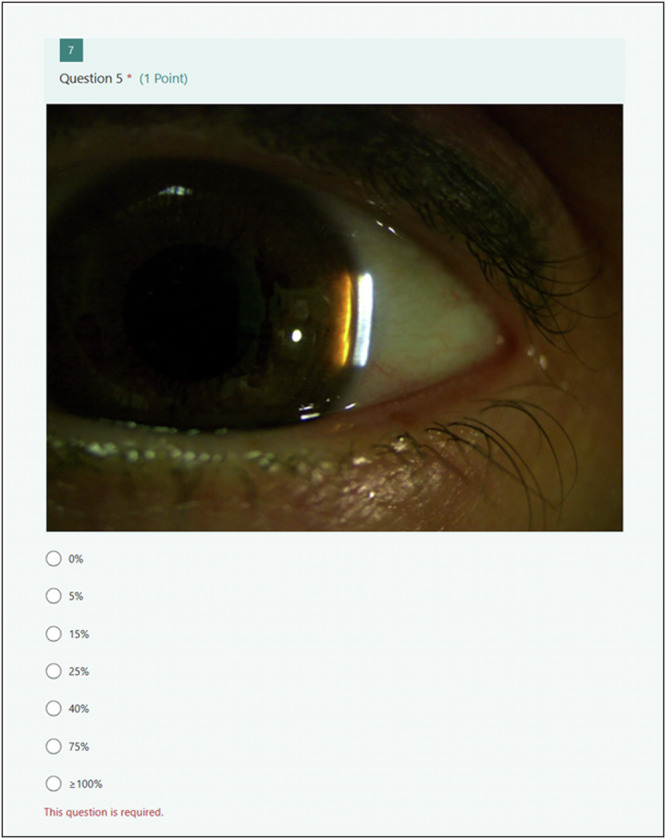
Vignette example from the assessment for users to grade the limbal anterior chamber depth.

Recruited optometrists were provided with an online link to access the first assessment (Assessment 1), where they inputted their study number and were provided with instructions and an example vignette before they undertook the assessment. Users then graded each LACD image using the 7-point percentage grading scale [12], using a forced choice method in one sitting (Fig. 1). After completing the assessment, they were then provided with another link to access the education package that was held on the MEH learning portal and progress was recorded via NHS secure servers.

The education package consisted of a 1.5-h recorded lecture on primary angle closure disease that contained multiple-choice questions and links to relevant publications. The lecture covered glaucoma classification, PACG mechanisms, PACG risk factors, tests for case finding and diagnosis alongside their accuracy, LACD interpretation, LACD application, evidence-based management of primary angle closure and guidelines on referral.

After the education package, they completed a 2nd online assessment (Assessment 2) in one sitting, using the same case mix and grading scale as the first assessment. The second assessment images consisted of contralateral eyes from the first assessment. The order of vignettes for both assessments was randomised by a random number generator.

The assessments and education package were independently piloted for clarity, assessment and layout by a learning and technology specialist and an optometrist. Completion of each assessment and the education package was tracked and logged electronically for all participants between August 2023 to January 2024. Progress reminders were sent monthly to all participants to complete the relevant stages of the study.

### Statistical analysis

For the assessment, a minimum number of 66 images was required for each assessment to produce a significant kappa value ≥0.4 at a significance level of 0.05 with 80% power [16]. For the number of optometrists, there was no formal sample size calculation, however in two recent vignette studies, using a similar computer-based assessment, a sample of 53 optometrist graders provided sufficiently narrow confidence intervals [17, 18].

The level of agreement between observers was calculated using linear weights for the kappa statistic for both sets of images using the 7-point scale as well as the traditional van Herick grades 1 to 4 [11], where the percentages were respectively converted. The weighted kappa was calculated by comparing the grade recorded by each participant against the grade determined by the expert panel. The weighted kappa attaches greater significance to larger differences between ratings than smaller differences. Landis and Koch proposed the following as standards for strength of agreement for the kappa statistic; <0 = poor, 0.00–0.20 = slight, 0.21–0.40 = fair, 0.41–0.60 = moderate, 0.61–0.80 = substantial and 0.81–1.0 = almost perfect [19].

Sensitivity and specificity were calculated pre and post-educational intervention by using LACD thresholds of ≤25% (grade 2 and less) and <25% (grade 1), where the assigned expert panel grade was used as the reference standard, respectively. The LACD threshold ≤25% was selected from the Cochrane meta-analysis that demonstrated it’s potential for case-detection of occludable angles [4] and LACD < 25%, which is recommended in current guidelines [7, 8].

All statistical analyses was performed using Medcalc (v22.0, Medcalc Software http://www.medcalc.org) and SPSS software (v29.0, IBM Inc., USA). Parametric and non-parametric tests were undertaken, where relevant. For the subgroup analysis, ANCOVA was used to determine any differences pre and post-educational intervention. For all inferential statistical tests, a *p* value of <0.05 was considered statistically significant.

## Results

### Assessment database

One hundred and twenty patients consented to participate in the study. Twenty patients were excluded as their images could not be captured; 13 left due to time constraints, 6 had poor fixation and 1 patient had poor imaging due to equipment failure.

Two hundred images were graded by the expert panel. One hundred and eighty-four images were allocated a grade through majority agreement, 14 images were graded independently using a graticule and two images were found to be uninterpretable by all three graders.

Pairwise comparisons between the expert panel observers using a mean weighted kappa for the 7-point grading scale were fair to substantial (0.39–0.66). When the grades were converted to the 4-point scale, interobserver agreement was substantial to almost perfect (0.61–0.81).

Eighty-four images were selected for each assessment that met the case-mix criteria of 20% narrow angles. Full details of the case mix and relevant participant details that were used for the assessments are displayed in Table 1.

**Table 1:** Case-mix and participant demographics of assessments.

	Assessment 1	Assessment 2
Mean age of all participants (95%CI)	56.1 (52.7–59.6)	56.4 (53.0–59.9)
Female (No. %)	56 (66.6%)	52 (61.9%)
	**Assessment 1(No. %)**	**Assessment 2 (No. %)**
Number of right eyes	44 (52.4%)	41 (48.8%)
LACD grade		
5%	2 (2.4%)	2 (2.4%)
15%	6 (7.1%)	7 (8.3%)
25%	9 (10.7%)	8 (9.5%)
40%	11 (13.1%)	11 (13.1%)
75%	17 (20.2%)	17 (20.2%)
100%	39 (46.4%)	39 (46.4%)
Total	84 (100%)	84 (100%)

### Optometrist demographics

Fifty-seven optometrists completed the online questionnaire that asked questions about their professional status. Amongst them, 52 completed the first online assessment (Table 2) and 49 (94.2%) completed both the assessments and the education package. Just under a quarter of optometrists 12 (23.1%) worked within secondary care, where 6 (11.5%) had reported working in a dedicated glaucoma clinic. A total of 35 optometrists (67.3%) had either formal glaucoma post-graduate CoO qualifications or had undertaken continued professional development (CPD) related to glaucoma. Of the 25 with CoO qualifications, 24 had the professional certificate in glaucoma and one had the diploma in glaucoma. One optometrist had independent prescribing status and a professional certificate in glaucoma. Further findings from the questionnaire have been described elsewhere [10].

**Table 2:** Professional qualifications, and demographics of optometrists who completed the first online assessment.

	Median (IQR)	*n*	%
A. Number of years qualified	19.0 (9.3–24.8)		
B. Gender			
Male		30	57.7
Female		22	42.3
C. Setting of primary practice			
Independent		33	
Multiple		10	
Locum		9	
D. No. optometrists working in secondary care		12	
- No. days working in secondary care in a week	3.5 (1.5–5.5)		
E. Optometrists working in glaucoma referral refinement/repeat measures scheme		28	
F. Optometrists with glaucoma post-graduate qualifications (College of Optometrists)		25	
G. Attended training/courses that discuss primary angle closure detection/management			
- WOPEC Glaucoma		6	
- CPD lectures		4	
- Independent prescribing post-graduate modules		1	

*CPD* continuous professional development, *WOPEC* Wales Optometry Post-graduate Education Centre.

### LACD interobserver agreement pre and post-education intervention

Interobserver agreement between CBO and the ophthalmologist’s reference grade using a mean weighted kappa for the 7-point LACD grading scale for assessment 1 was 0.42 (Moderate), 95% CI 0.36–0.48 (Slight-moderate). Following the education intervention for assessment 2, the mean increased to 0.47 (Moderate) 95% CI 0.42–0.53 (moderate) Table 3).

**Table 3:** Sensitivity and specificity of LACD grades <25% and ≤25% for pre and post-education intervention.

	Sensitivity Grade 1 LACD threshold <25% Mean 95% (CI)	Specificity LACD threshold <25% Mean 95% (CI)	Sensitivity LACD threshold ≤25% Mean 95% (CI)	Specificity LACD threshold ≤25% Mean 95% (CI)
Assessment 1	86.0 (79.9–91.3)	84.6 (82.3–86.9)	92.1 (88.0–96.1)	83.6 (80.6–86.7)
Assessment 2	78.2 (71.4–85.1)	90.4 (88.1–92.8)	81.2 (75.8–86-6)	85.0 (82.0–88.1)
*p* value	0.049	<0.001	<0.001	0.24

For the 4-point grading scale, the mean weighted kappa for assessment 1 was 0.61 (Substantial), 95% CI 0.56–0.66 (moderate-substantial) and for assessment 2 it was higher at 0.64 (Substantial), 95% CI 0.58–0.69 (moderate-substantial).

### LACD accuracy pre and post-education intervention

The mean sensitivity pre-education intervention for optometrists identifying a LACD threshold of <25% was 86.0% 95% CI (79.9-91.3), which decreased by 7.8% post-education intervention and mean specificity increased by 5.8% to 90.4% (88.1–92.8), where both were statistically significant (*p* < 0.001). The mean sensitivity pre-education intervention for optometrists identifying a LACD threshold of ≤25% was 92.1% (88.0–96.1), which decreased by 10.9% post-education intervention (*p* < 0.001) and mean specificity increased by 1.4% but this was not statistically significant (Table 3).

### LACD accuracy subgroup analysis

Mann–Whitney analysis within assessment 1 for those who had or did not have glaucoma qualifications for grade <25% found there was no statistically significant difference in sensitivity for both groups but there was a statistically significant improved performance in specificity (*p* = 0.004) for those who had a glaucoma qualification. For grade ≤25%, those with a glaucoma college qualification were found to have a lower sensitivity (*p* = 0.016) but achieved higher specificity (*p* = 0.001) compared to those without. Analysis within assessment 2 found no statistically significant differences in performance for grades <25% and ≤25% in terms of sensitivity and specificity between those who had or did not have glaucoma qualifications.

Subgroup analysis evaluating variances in accuracy for grades <25% and ≤25% found there was no difference amongst genders or between those working and not working in a glaucoma referral/refinement scheme for both assessment cohorts (within assessments).

ANCOVA analysis pre and post-education intervention (between assessments) evaluating subgroups concerning glaucoma qualifications or if they working within a glaucoma refinement/repeat measures scheme found no difference in performance for grades <25% and ≤25%

## Discussion

To our knowledge, this is the first study that has evaluated interobserver agreement of community-based optometrists using the 7-point LACD grading scale and whether an education intervention would affect the measurement of LACD. Our key findings suggest that an educational intervention improved interobserver agreement between optometrists and ophthalmologists when interpreting LACD. Agreement for the 4-point grading scale both pre and post-education intervention was higher than pre or post-education intervention of the 7-point scale. It was also found that an education intervention improved specificity when using the clinical guidelines recommended threshold (<25%) for further referral, but decreased sensitivity, where both were statistically significant.

Optometrists based in the United Kingdom, working in primary care, have a key role in detecting those at risk of glaucoma and are one of the main sources of referral to tertiary centres [20, 21]. Current professional guidance on the management and referral of primary angle closure [7, 8] includes referral criteria for CBO, that incorporates a referral LACD threshold of <25%, which yields a relatively high sensitivity and specificity for an occludable angle using gonioscopy [4]. Interobserver agreement of this technique amongst clinicians who actively work within an ophthalmology glaucoma setting is substantial, with kappa scores ranging from 0.60–0.80 [9, 12, 22]. However, there is a paucity of data evaluating this technique with CBO. Our findings found that interobserver agreement of the LACD amongst CBO was substantial (pre-education intervention, mean Kw 0.61) using the traditional 4-point grading scale, compared to a similar study which found moderate agreement (mean Kw 0.50) [9]. The case mix between the two studies was relatively similar, where 20-25% of images included narrow angles (<25%). However, the proportion of those who had undertaken accredited post-graduate qualifications was higher in the current study. This may have impacted the overall findings as almost half of the cohort had attained the College of Optometrists accredited post-graduate professional certificate in glaucoma. This qualification has been recommended for optometrists to undertake if they are to participate in glaucoma-enhanced referral schemes; as recommended by the NICE-approved joint RCO and the Clinical Council for Eye Health Commissioning Guide: Glaucoma [23].

The Royal College of Ophthalmologists has recommended further upskilling and support of healthcare professionals [24] that could include undertaking comprehensive post-graduate training and/or targeted training for specific tasks [25–29], where education interventions have been reported to be beneficial in the interpretation of glaucoma- related diagnostic tests [17, 18, 30]. It has been found that the accuracy of PAC referrals by CBO range from low to moderate [5, 6], where it has been hypothesised that the adoption of finer grading scales and training could enhance LACD test performance and accuracy [9]. Furthermore, a recent study found that despite the joint guidance publication for PAC referral, guidance uptake was moderate amongst CBO and the authors recommended that there should be further training regarding PAC management in primary care [10]. As far as the authors are aware, this is the first published report evaluating interobserver agreement between CBO and ophthalmologists using the 7-point LACD grading scale. We report that there was moderate interobserver agreement (mean Kw 0.42) which was lower than the 4-point scale. This could be attributed to the difference in the number of categories, despite using linear weights to minimise the effect of this [31]. In the current study, an education intervention was utilised that demonstrated an improvement in the kappa value for both grading scales. The education intervention improved specificity but lowered sensitivity, which was statistically significant when using the recommended joint guidance threshold for referral across the whole cohort. Whilst there was a drop in sensitivity, the prevalence of primary angle closure glaucoma in the European population is relatively low [1, 32]. Therefore efforts should be directed to maximise specificity to identify those at highest risk of PAC to reduce the impact of false positive referrals. This in part was one of the drivers of the joint guidance regarding PAC referral to improve specificity through the ‘PAC suspect plus’ criteria that combined a referral threshold with a risk factor for this disease to identify those at higher risk. Furthermore, subgroup analysis of the cohort when comparing CBO with and without the glaucoma qualifications found there was a difference in specificity when utilising the LACD grade <25% pre-education intervention, yet post-intervention performance was similar in both sensitivity and specificity. This is a tantalising finding, regarding LACD accuracy, the provision of a relatively quick and accessible education package could be made available with relatively little effort in terms of time and cost to both the participant and glaucoma provider [17, 18, 30]. In addition, this education intervention could be rapidly deployed into existing CBO referral pathways and routine practice that would align with Royal College of Ophthalmologists recommendation and supplement efforts to reduce the number of PAC false positive referrals to the hospital eye service.

Strengths of this study include that the images acquired were from real-world new at-risk glaucoma patients that are applicable to those recently referred from primary care, to ascertain optometric accuracy. The case mix for both assessments was equivalent and encompassed a range of LACD’s, where the reference grading was undertaken by masked consultant ophthalmologists who specialised in glaucoma. In addition, all the participants were working in primary care across a range of settings where the findings would be applicable and only a minority worked in secondary eye care [33]. The study’s main aims were to determine interobserver agreement and accuracy of the LACD threshold between CBO and ophthalmologists. The vignettes did not include supplementary information relating to the patient’s history where referral accuracy could be further explored, nor can they represent a dynamic LACD slit-lamp examination. Furthermore, the reference standard used to calculate sensitivity and specificity was not gonioscopy, therefore these indices relating to the detection of the primary angle closure spectrum [34] can’t be compared. In addition, almost half of the optometrists who were recruited had post-graduate qualifications in glaucoma and/or they worked in a glaucoma referral refinement/shared care scheme that is not representative of those working in primary care [35, 36], therefore this may affect the applicability of our findings.

In conclusion, it is known that most glaucoma referrals are initiated by CBO where the accuracy of PAC referrals and knowledge of primary angle closure management is moderate. The results of our study imply the use of the 4-point LACD grading scale when referring for further examination instead of the 7-point grading scale. Exploration of an accessible online education intervention showed improved interobserver agreement between optometrists and ophthalmologists as well as specificity using the joint referral guidance threshold for PAC, respectively. This intervention might reduce false positive PAC referrals and could be rapidly disseminated to those working in primary care. Further research is required to determine whether such an intervention would affect real-world primary angle closure referrals by CBO.

Supplemental material is available at Eye’s website.

## Summary

### What was known before

- Accuracy of referrals for suspected primary angle closure (PAC) by community-based optometrists (CBO) to the hospital eye service is low to moderate.
- CBO interobserver agreement of limbal anterior chamber depth and application of clinical guidelines for the referral of PAC to the hospital eye service are both moderate.

### What this study adds

- The 4-point LACD grading scale would be more applicable rather than the 7-point scale in a case-finding setting.
- An accessible education package improved CBO’s agreement of LACD and specificity using the joint Royal College of Ophthalmologists/College of Optometrists PAC referral threshold (LACD less than 25%, (grade 1)).
- This education package may reduce false positive PAC referrals and could be rapidly disseminated to CBO’s. Further research is needed to assess real-world PAC referrals after an education intervention.

## Supplementary information

Survey of clinical decisions of those at risk of primary angle closure by community optometrists

## Data Availability

The datasets generated from the current study are not publicly available due to data use agreements with MEH. Requests for data will require approval from MEH and the signing of data access agreements; requests can be submitted to the corresponding author.
