# Correction: An education-based intervention investigating the accuracy of community-based optometrists evaluating limbal anterior chamber depth

**DOI:** 10.1038/s41433-025-03810-5

**Published:** 2025-04-25

**Authors:** Anish Jindal, Tess Agnew, Dilani Siriwardena, Eleni Nikita, Winifred Nolan

**Affiliations:** 1 Moorfields Eye Hospital NHS Foundation Trust, London, UK; 2 Institute of Ophthalmology, University College London, London, UK; 3 NIHR Moorfields Biomedical Research Centre, London, UK

**Keywords:** Education, Health care

Correction to: *Eye* 10.1038/s41433-024-03492-5, published online 6 December 2024

The article “An education-based intervention investigating the accuracy of community-based optometrists evaluating limbal anterior chamber depth”, written by Anish Jindal, Tess Agnew, Dilani Siriwardena et al., was originally published under exclusive license to “The Author(s), under exclusive licence to The Royal College of Ophthalmologists 2024”. As a result of the subsequent decision to publish the article under the open access model, the article’s copyright notice was changed on 31 March 2025 to “© The Author(s), 2025” and the article is now distributed under a Creative Commons Attribution 4.0 International License, which permits use, sharing, adaptation, distribution and reproduction in any medium or format, as long as you give appropriate credit to the original author(s) and the source, provide a link to the Creative Commons licence, and indicate if changes were made. The images or other third party material in this article are included in the article’s Creative Commons licence, unless indicated otherwise in a credit line to the material. If material is not included in the article’s Creative Commons licence and your intended use is not permitted by statutory regulation or exceeds the permitted use, you will need to obtain permission directly from the copyright holder. To view a copy of this licence, visit http://creativecommons.org/licenses/by/4.0/.

