# Supplementary material for: An education-based intervention investigating the accuracy of community-based optometrists evaluating limbal anterior chamber depth
Source: Eye (Lond). 2024 Dec 6;39(5):963–8. doi: 10.1038/s41433-024-03492-5 (PMC11933265; doi:10.1038/s41433-024-03492-5)
Supplement: Supplementary file 1 — Survey of clinical decisions of those at risk of primary angle closure by community optometrists [file 41433_2024_3492_MOESM1_ESM.pdf]

Evaluation of clinical decisions and assessment of those at risk of primary angle closure by community optometrists: Stage 2

Evaluation of clinical decisions and assessment of those at risk of primary angle closure by community optometrists: Stage 2

The following questionnaire will ask about your mode of practice, prior training and clinical decision making with respect to examining and referring patients at risk of primary angle closure. This survey will take approximately 30 minutes, your responses will remain confidential. If you have any queries or problems with the survey, please email a.jindal@nhs.net.

Many thanks for taking the time to complete the survey, your participation is much appreciated.

---

1) Please input your Study Number

____

---

2) Which of the following is your principal mode of community practice?

- ◯ Independent
- ◯ Multiple/Group
- ◯ Locum
- ◯ Other

---

3) Do you work in secondary care (Hospital)?
-If no, please go to question 5.

- ◯ Yes
- ◯ No

---

4) During the last working month how many days in the week did you spend working in the HES?

◯ 1 ◯ 2 ◯ 3 ◯ 4
◯ 5 ◯ 6 ◯ 7

---

5) In the hospital, do you work in a glaucoma clinic?

◯ Yes ◯ No

---

6) Are you working/involved in a glaucoma community shared care or glaucoma referral refinement pathway ?

◯ Yes ◯ No

---

7) Have you completed any College of Optometrist Glaucoma postgraduate qualifications i.e professional/higher certificates/diploma in glaucoma.
-If no, please go to question 9

◯ Yes ◯ No

---

8) If you have completed any glaucoma postgraduate qualifications, please can you provide details.

____

---

9) If you have attended any other training or lectures that have discussed primary angle closure detection/management that are not related to the College glaucoma qualifications, please can you detail

____

---

10) Are you aware of any risks factors associated with primary angle closure? If so, please can you list them.

____

---

11) When you examine adult patients, what tests would you carry out before you refer a person to the Hospital Eye Service that you suspect with primary angle closure/primary angle closure glaucoma? Please detail

____

12) From your clinical experience, when you have referred patients with suspected primary angle closure/glaucoma, have most of your referred patients reported any symptoms associated with angle closure?

◯ Yes ◯ No

13) When referring asymptomatic patients based on van Herick alone and without risk factors, what would be your threshold for referral? Please select one answer.

◯ 0% ◯ ≤5% ◯ ≤15%
◯ < 25% (grade 1) ◯ ≤25% (grade 2 or less) ◯ 25% (grade 2)
◯ ≤40% ◯ >25% to < 100% (grade 3)
◯ I wouldn't refer

14) When referring asymptomatic patients for primary angle closure, do you take into account any of the risk factors that you have detailed earlier?

◯ Yes ◯ No

15) When referring asymptomatic patients based on van Herick with risk factors, what would be your threshold for referral? Please select one answer.

◯ 0% ◯ ≤5% ◯ ≤15%
◯ < 25% (grade 1) ◯ ≤25% (grade 2 or less) ◯ 25% (grade 2)
◯ ≤40% ◯ >25% to < 100% (grade 3)
◯ I wouldn't refer

16) Please detail any guidelines you use when examining or referring patients whom you suspect of primary angle closure?

____

17) Are you aware of The Zhongshan Angle Closure Prevention Trial (ZAP study)?

◯ Yes ◯ No

18) Are you aware of the effectiveness of early lens extraction for the treatment of primary angle-closure glaucoma (EAGLE trial)?

◯ Yes ◯ No

19) Are you aware of the Cochrane systematic review on non-contact tests detecting those at risk of primary angle closure glaucoma?

◯ Yes ◯ No

20) Clinical Scenario

A 64-year-old Caucasian patient with no associated angle closure risk factors was initially referred for primary angle closure investigation. They were subsequently diagnosed by the hospital eye service (HES) as a primary angle closure suspect where they found 180 degrees of irido-trabecular contact on gonioscopy in both eyes, IOP of 15mmHg in both eyes, healthy fields and retinal nerve fibre layer and no signs of glaucomatous optic neuropathy. No medical or surgical intervention was provided and they were subsequently discharged back to your primary care practice for monitoring, please answer the following questions (a-d):

a. How often would you review them?

____

21) b. What tests would you conduct when you are monitoring them?

____

22) c .Please rate your confidence in monitoring them (1 not confident, 10 fully confident)
◯ 1 ◯ 2 ◯ 3 ◯ 4
◯ 5 ◯ 6 ◯ 7 ◯ 8
◯ 9 ◯ 10

23) d. If you were to re-refer them back to the HES, what findings would you refer them on? Please detail
____

24) Do you have access to fundus photography in the community?
◯ Yes ◯ No

25) Do you have access to OCT in the community?
◯ Yes ◯ No

26) Do you have access to Anterior Segment OCT in the community?
◯ Yes ◯ No

27) Have you received any training specific to AS-OCT interpretation?
◯ Yes ◯ No

28) Can you perform gonioscopy?
◯ Yes ◯ No

29) Do you use gonioscopy in the community?
◯ Yes ◯ No

30) In which year did you initially register with the GOC?
____

31) What gender do you identify as
◯ Male ◯ Female ◯ Trans-gender
◯ Non- Binary ◯ Other
◯ Prefer not to answer
